# Breastfeeding: Nature’s Safety Net

**DOI:** 10.5005/jp-journals-10005-1133

**Published:** 2012-02-24

**Authors:** Manisha Agarwal, S Ghousia, Sapna Konde, Sunil Raj

**Affiliations:** Reader, Department of Pedodontics and Preventive Dentistry, AECS Maaruti College of Dental Sciences and Research Centre, Bengaluru Karnataka, India, e-mail: drmani29@gmail.com; Senior Lecturer, Department of Pedodontics, AECS Maaruti College of Dental Sciences and Research Centre, Bengaluru, Karnataka, India; Professor and Head, Department of Pedodontics and Preventive Dentistry, AECS Maaruti College of Dental Sciences and Research Centre, Bengaluru, Karnataka, India; Professor, Department of Pedodontics and Preventive Dentistry, AECS Maaruti College of Dental Sciences and Research Centre, Bengaluru Karnataka, India

**Keywords:** Breastfeeding, Anticipatory guidance, Orofacial growth and development

## Abstract

Breastfeeding is a natural safety-net for the first few months in order to give the child a fairer start to life. The American Academy of Pediatric Dentistry recognizes the distinct nutritional advantages of human milk for infants and endorsed the position of the American Academy of Pediatrics on the promotion of breastfeeding. It therefore calls for increase in need to negotiate the roles and responsibilities of pediatric dentists to eliminate the existing gaps in preventive care and anticipatory guidance. The objective of this evidence-based review is to explore the beneficial roles of breastfeeding in orofacial growth and development and endorse the same through anticipatory guidance.

**How to cite this article:** Agarwal M, Ghousia S, Konde S, Raj S. Breastfeeding: Nature’s Safety Net. Int J Clin Pediatr Dent 2012;5(1):49-53.

## INTRODUCTION

Life in its fullness is enjoyed when mother nature is obeyed!

                                                                                 *– Weston Price*

Dental caries and malocclusion related to abnormal oral habits are the most prevalent conditions affecting infants and children. Pediatricians and pediatric dentists coincide in the great importance of breastfeeding during the first 6 months of life for the correct development of the mouth and for occlusion, breathing and swallowing during childhood.

The American Academy of Pediatric Dentistry (AAPD) considers breast milk an ideal nutrition as it provides innumerable health-related advantages to infants, mothers and society. WHO recommends maintaining breastfeeding up to the second year of life or longer. Breastfeeding is usually recommended by healthcare professionals and pediatricians to be continued as long as mutually desired by mother and child.^1^

Breast milk is characterized by complex constituents like IgA antibodies, oligosaccharides, lactoferrin and hormones that may confer immunity to the infant. Human milk remains the single most important nutritional and bioactive substance available to the neonate. Breastfeeding also remains the first and best way to form a secure bond between mother and child, nurturing communication and emotional development.^2^

Oxytocin released during breastfeeding may provide a biological basis for human attachment and bonding. The mixture of nutritional factors and growth hormones in human milk has been linked to enhanced cognitive development.^2,3^

It also helps to develop a correct positioning of the dental arches and therefore a good dental occlusion. This manuscript therefore aims to address the following concepts:

- The role of breastfeeding in orofacial development and prevention of malocclusion.
- Protective role of exclusive breastfeeding during the first 6 months of life against dental caries, enamel fluorosis and SIDS (sudden infant death syndrome).
- Prevention of cancer
- Emphasizing breastfeeding through anticipatory guidance.

### History—A Breastfeeding Tale^4^

Breastfeeding has been important since the beginning of mankind. In Sparta, Greece, Spartan women were required to nurse their eldest son. This was the child who was to inherit the family name. At other times, breastfeeding has been something that only lower class people did, and wet nurses were employed to breastfeed the children of the royal families.

### Taboos Related to Breastfeeding

The upper class society considered breastfeeding as unclean and not a well-accepted social norm. Inadequate milk secretion, subsequent pregnancies, maternal illness or medications, use of oral contraceptives, return to work, were among the few other reasons for breastfeeding to become unpopular. It was once considered to be act of crime and offence in certain parts of the world like UK, Scotland, England and America. In India, according to some Brahminical literature, breastfeeding was practiced in 2nd century only after the fifth day of child’s birth, allowing the colostrum to be discarded and the true breast milk to flow.

### There and Back Again

Once thought to be a passé, breastfeeding has been re- discovered by modern science as a means to save lives, reduce illness, foster optimum development and protect the environment.

In 1970s, pendulum swings back – women get back to earthy ways of infant feeding. Policy makers are increasingly recognizing that breastfeeding promotion efforts can reduce healthcare costs and enhance maternal and infants well- being, decrease the risk of congenital anomalies or infant’s cancer.

### La Leche League (LLL) Organization

La Leche means ‘the milk’ in Spanish. Initiated by Dr Grantly-Dick Read, this organization has been instrumental in worldwide promotion of breastfeeding for the last four decades since 1957 and by 1974, it was accredited by the American Medical Association to provide continuing educational credits to healthcare professionals regarding womanly art of child feeding. It has been fighting successfully in gaining lactation periods and lactation rooms at lactating mother’s work place.

In 1979, LLL International (LLLI) was represented at Joint WHO/UNICEF Meeting on Infant and Young Child Feeding in October in Geneva, Switzerland. In 1981 - LLLI establishes affiliate status as part of UNICEF’s organizational structure. LLLI joins with other organizations and individuals to form the World Alliance for Breastfeeding Action (WABA), a global network to protect, support and promote breastfeeding in 1991 and in 1992 WABA sponsored first *World Breastfeeding Week*, *August 1-7*, with the theme: Baby-Friendly Hospitals.^4^

### Position of the AAPD on Breastfeeding in 1996

- The risk of potentially devastating nursing-pattern dental decay exists for the breastfed child as it does for the bottlefed child.
- Ad libitum nocturnal breastfeeding should be avoided after the first primary tooth begins to erupt.

### Anthropology Deciduous Caries Data on Prehistoric Infant Skull—1997

The data obtained from the research work of Dr Brian Palmer states that breastfed babies had no decay for 92,000 years. Moreover, out of 4,640 mammals all of whom breastfed their infants, only human beings have dental decay. The skull forms of breastfed infants showed a wider palatal arch, good width between the pterygoid plates, nice facial forms and most importantly, huge large nasal openings or chonchae which are less likely to collapse. The conclusion was that breastfeeding was the only way of nurturing infants in the past, the skulls of whom, exhibit minimal decay and rare malocclusions and it causing dental decay was based only on population-based studies, rather than laboratory studies.^5^

Dr Pamela Erickson in the year 1999, at the University of Minnesota, concluded that breast milk is not cariogenic but when sugary substances are attached with breastfeeding; breast milk becomes a dangerous catalyst that can lead to rampant caries. Her research further concluded that breast milk prohibits acid and bacterial growth in the mouth. However, breast milk has a ‘low buffering capacity’ and does not buffer the addition of acid. When breast milk is alternated with sugar, the rate of caries development is faster than that of sugar alone.^6^

### Guilt of Association AAPD—1999

The association confessed that the accusation of infant dental decay was made initially in the year 1977 based on only four case reports which were from a single population group. Authors of that study felt they were the first to make the link between breastfeeding and caries: ‘There are no notation in the dental literature about breastfeeding and its relationship to dental caries’. This article is referenced in many other articles accusing breastfeeding as a causative factor for infant caries.

### American Academy of Pediatrics (AAP) Breastfeeding Recommendations (2005)

Exclusive breastfeeding is sufficient to support optimal growth and development for approximately the first 6 months of life. Breastfeeding should be continued for at least the first year of life and beyond for as long as mutually desired by mother and child.

### AAP Condemn and Warnings on Babywise Feeding/Parent Directed Feeding—1998, Revised in Jan 2010

Breastfeeding on a parent-determined schedule including a ‘flexible routine’ is called in Babywise feeding.

The AAP has stated, ‘the best feeding schedules are the ones babies design themselves. Scheduled feedings designed by parents may put babies at risk for poor weight gain and dehydration’. It has also been associated with failure to thrive (FTT), poor milk supply and involuntary early weaning.

### WHO Breastfeeding Recommendations (2007)

Exclusive breastfeeding for 6 months is the optimal way of feeding infants. Thereafter infants should receive complementary foods with continued breastfeeding up to 2 years of age or beyond.

### AAPD Statement in 1999, Revised in 2010

Breast milk alone does not cause tooth decay! ‘exclusive breastfeeding’ does not mean that the infant will be immune from decay. Human breast milk is not cariogenic!

### Process of Breastfeeding^7^

In breastfeeding, the baby pulls and sucks the nipple into the mouth. Part of the areola is also held in the mouth and so nipple is held as far back as the junction of hard and soft tissue palates. The lips form a seal and the mouth cavity is enlarged as the jaw moves. The whole of the lower jaw is raised and lowered alternately with a rocking motion. The tongue is protruded and remains in contact with the lower lip throughout. As the jaw is lowered, the body of the tongue moves downwards and forward. In X-ray films, this has been described as looking like a boat rocking upon waves.

The nipple is considerably extended and taken well back into the mouth, and the squeezing action is completed by the contraction of the floor of the mouth.

### Review of Breastfeeding Benefits

Breastfeeding offers many benefits to the baby and the mother. Breast milk provides the right balance of nutrients to help an infant grow into a strong and healthy toddler, as it provides all the essential ingredients needed for growth and development. It increases the serotonin receptors in the brain and enhances the IQ development of the child. It ultimately optimizes mother-infant bonding.

Breastfeeding reduces the risks of otitis media, asthma, urinary tract infections, gastroenteritis, allergies, child obesity, cancer/childhood lymphomas, bacteremia, meningitis, botulism, juvenile diabetes, ulcerative colitis, Crohn’s disease, childhood leukemia and CVS disorders in a child.^8^

Prevention of SIDS

Breastfeeding is important to the proper development of the swallowing action of the tongue, proper alignment of the teeth and the shaping of the hard palate. It also reduces the frequency in victims of child abuse and neglect along with reduction in incidents of SIDS or ‘crib death’. Breastfed infants rarely suffer from obstructive sleep apnea (OSA) and long face syndrome.^9^

Orofacial Growth and Development and Prevention of Oral Habits

The control of the growth involves a complex interaction with local functions responding to local signals, which must act in concert with other regions. Growth is strongly influenced by genetic, functional and environmental factors. Breastfeeding has an effect on oral musculature function and thereby have an effect on craniofacial and dental development. Malar growth was found to increase with increasing duration of breastfeeding. Infants that have been breastfed for more than 3 months have the best malar growth.^10^ Breastfeeding also lowers the risk of the antero- posterior malrelationships. The breastfed infants showed the least amount of relative change in maxillary arch length and palatal depth. It has been found that breastfeeding encourages correct intermaxillary relationship. It was also observed that breastfed infants have the lowest prevalence of digital habits also a direct relationship between length of breastfeeding and occlusion, the longer the infant was breastfed, the better was the occlusion.^11^-^13^

On the contrary, bottlefeeding leads to a habit of forward tongue-thrusting and a weakened development of orbicularis muscles. Anything placed in a child’s mouth excessively other than the mother’s breast can impact occlusion. The impact is affected by a number of factors, including intensity, duration and frequency. While the soft breast adapts to the shape of the infant’s mouth, anything firm requires the mouth to do the adapting.^14^ In addition, during breastfeeding, the tongue moves in a peristaltic motion underneath the breast. This motion is critical for the proper development of swallowing, alignment of the teeth and the shaping of the hard palate.^15^-^17^ Movement of the tongue is also a reason for clipping a tight lingual frenulum in a newborn. This will allow the tongue to compress the breast and to develop the proper motion. By preventing this motion, a tight frenulum can lead to a tongue thrust with a resultant malocclusion.^18^-^20^

Prevention of Dental Decay

Breastfeeding of the child for more than 40 days may act preventively and inhibit the development of nursing caries in children. Human milk does contain lactose (milk sugar), which is a potential carbohydrate food source for the cavity causing bacteria, but the immune boosting properties of breast milk seem to over ride this. The anti-infective activity of human milk is potentially greater than the sum of its microbicidal components like secretory IgA, lactoferrin, Lewis factor X, SLPI (secretory leucocyte protease inhibitor), ***a ***defensins, complements, mucins, prostaglandins and interleukins. (CE Isaacs, 2004).

Breast milk therefore protects against all infectious diseases of infancy, including dental caries.^21^

*‘Lactose’ in breast milk-False accuse for caries *Lactose sugar is present in breast milk but it is not easily fermented as sucrose. It is naturally digested and broken down into sugars while in lower intestine and not in mouth. Lactic acid promotes *Lactobacillus bifidus. L. bifidus *helps prevent intestinal putrefaction. Cariogenic bacteria may not utilize lactose as an energy source as readily as sucrose.

*Streptococcus mutans *is highly susceptible to the bactericidal action of lactoferrin. Lactoferrin chelates iron, making this essential nutrient inaccessible to an invading microorganism. The human milk components are able to inhibit adhesion of *S. mutans *to hydroxyapatite crystals.^22,23^

Prevention of Fluorosis

Formula-fed children had tap water added to the bottle. Breastfeeding for >6 months may protect children from developing fluorosis in the permanent incisors.^24^

Prevention of Cancer

Human breast milk kills cancer cells. The substance known as HAMLET (human alpha-lactalbumin made lethal to tumor cells) - the cancer killer is one of the most abundant proteins in breast milk. It helps to produce lactose. Acids in the stomach are the key to its activation.^25^

### Benefits of Breastfeeding in the Infants with Special Needs^7^

Down’s Syndrome

The infant will be affected with hypotonia (weak suck, poor head control, tongue protrusion, poor lip closure). The breastfeeding can be followed with normal tongue placement. This strengthens jaw muscles which are used to coordinate sucking and swallowing.

Congenital Heart Disease

This infant requires more frequent feedings with upright or semi-upright positioning. Mothers must be aware and warned of signs of fatigue during feedings increased respiratory efforts, sweating and falling asleep.

Cleft Lip-Palate Infants

Unfortunately complete clefts preclude feeding, as breast feeding is not possible in these infants. A soft, large bottle with large hole is required and a palatal prosthesis may be required. In infants affected with incomplete cleft/cleft lip, a dancer hold position; or modified football hold positioning is necessary to carry out breastfeeding.

Breastfeeding the Infant after Cleft Repair

Infants are discouraged from sucking for some time post operatively. Immediate postoperative feeding may be via spoon, syringe or a squeeze bottle.^26,27^

## CONCLUSION

In this review, it is emphasized that pediatric dentists should motivate lactating mothers in their efforts to increase the rate and duration of breastfeeding through anticipatory guidance. Breastfeeding of the child for more than 40 days may act preventively and inhibit the development of nursing caries in children and simultaneously acts positively in promoting orofacial growth and development. It also has beneficial effects in prevention of SIDS, fluorosis and to certain extent cancer. Weighing its beneficial effects, it is time for us to collaborate with pioneers of medical fields like pediatricians and other physicians to solve problems of joint interests.

The mouth is the window to many body systems, so there is increase in demand for efforts by pediatric dentists to play a key role in not only oral health of a child but its over all well being in general. This marks the first step in implementing breastfeeding through anticipatory guidance for infant health care needs.

Wayne Halstorm rightly quoted—‘Time has come to recognize that mouth is not divorced from the rest of the body; let’s put mouth back into the body!’
